# Glycosylation and the global virome

**DOI:** 10.1111/mec.16731

**Published:** 2022-10-21

**Authors:** Cassandra L. Pegg, Benjamin L. Schulz, Benjamin A. Neely, Gregory F. Albery, Colin J. Carlson

**Affiliations:** ^1^ School of Chemistry and Molecular Biosciences The University of Queensland St Lucia Queensland Australia; ^2^ National Institute of Standards and Technology Charleston South Carolina USA; ^3^ Department of Biology Georgetown University Washington District of Columbia USA; ^4^ Department of Microbiology and Immunology Georgetown University Medical Center Washington District of Columbia USA; ^5^ Center for Global Health Science and Security Georgetown University Medical Center Washington District of Columbia USA

**Keywords:** glycosylation, Microbial Biology, Modelling, Molecular Evolution, Viral Ecology, Viral Evolution

## Abstract

The sugars that coat the outsides of viruses and host cells are key to successful disease transmission, but they remain understudied compared to other molecular features. Understanding the comparative zoology of glycosylation ‐ and harnessing it for predictive science ‐ could help close the molecular gap in zoonotic risk assessment.

## 1 INTRODUCTION

Due to recent encounters with zoonotic viruses like Ebola virus and SARS‐CoV‐2, efforts to forecast the zoonotic risk of wildlife viruses ‐ and, more broadly, to understand the biological constraints on cross‐species transmission ‐ are increasingly appealing (Albery et al., 2021). To date, most of these efforts rely on easily‐observed traits of hosts, like morphology, diet, or phylogeny (Albery et al., 2020; Han et al., 2016). Despite their distance from the molecular determinants of transmission, these traits can be used to build models that have surprising predictive accuracy (Becker et al., 2022).

Many microbiologists have expressed a healthy scepticism of these approaches, which often entirely lack predictors that consider the molecular biology of hosts or any viral traits, and therefore, only coarsely infer the cellular processes of infection through other proxies that are correlated across evolutionary space. As a rare exception, genomic approaches are increasingly being used to close this gap (Babayan et al., 2018; Mollentze et al., 2021), and can help identify salient mechanisms of host‐virus interactions (e.g., CpG dinucleotide depletion in vertebrate viruses appears to help them evade innate immune responses like the zinc finger antiviral protein [Takata et al., 2017]). However, as predictive features, genomic traits are often confounded by evolutionary signals (Di Giallonardo et al., 2017; Shackelton et al., 2006), and genomic data only offer limited insights into the actual three‐dimensional structural compatibility of viral and host cell surfaces, a “lock‐and‐key” type process. This lock‐and‐key interaction not only allows efficient infection of susceptible hosts, but also limits cross‐species viral transmission. Structural modelling approaches have been used to examine the binding of viral proteins and host cell receptors, most recently in the context of research on SARS‐CoV‐2, but many of these simulations neglect key information: the glycosylation of these structures. Indeed, the handful that do address this aspect have revealed unexpected and important roles for glycosylation in these processes including those for SARS‐CoV‐2 (Casalino et al., 2020; Ghorbani et al., 2021; Sztain et al., 2021; Zhao et al., 2020), HIV (Berndsen et al., 2020; Ferreira et al., 2018; Lemmin et al., 2017; Stewart‐Jones et al., 2016; Wood et al., 2013; Yang et al., 2017), and influenza viruses (Kasson & Pande, 2008; Newhouse et al., 2009; Seitz et al., 2020; Xu et al., 2009).

The sugars, or glycans, that decorate host cell surface macromolecules are often critical ligands that viruses associate with to enter cells (Jones et al., 2021). Viral proteins from different virus families exhibit substantial variation in binding affinity towards host glycan receptors, and the compatibility between different viral proteins and host glycosylation varies between host and virus species, across tissues and organ systems, and even over time or between individuals (Jones et al., 2021; Maginnis, 2018; Thompson et al., 2019). Some viral surface proteins become glycosylated by host cell machinery during infection, and in the process can mimic host cell surfaces or shield proteins from antibody recognition, helping viruses evade the host's immune system (Bagdonaite et al., 2018; Bagdonaite & Wandall, 2018; Watanabe et al., 2019; Zhao et al., 2021). The glycans on viral surfaces are also recognized by host glycan‐binding proteins on immune cells that capture viruses, either preventing or promoting infection (Crocker et al., 2007; Erikson et al., 2015). These aspects of host‐virus compatibility can create or unlock barriers to transmission, but are poorly characterized as an underlying structural determinant of host‐virus networks because glycan structures are subject to rapid regulation and are sometimes perceived as being analytically challenging. We suggest an undertaking to describe the comparative zoology of glycoproteins, and their role in structuring the global virome.

## 2 WHAT SUGARS DO, AND HOW

Glycans are a key feature of the cell surface and extracellular matrix of eukarya, archaea, and bacteria (West et al., 2021). Glycans can be found on proteins or lipids, and technological advances (Everest‐Dass et al., 2018) in the 21st century have greatly increased our ability to identify, characterize, and manipulate glycosylation, which has in turn supported deeper insights into the multitude of diverse roles it plays (Moremen et al., 2012). Glycans can constitute a substantial proportion of the molecular mass of a protein (Varki, 2017), contributing to their biophysical properties and influencing protein targeting, folding, structure and secretion (Ohtsubo & Marth, 2006; Varki, 2017). Moreover, glycans are mostly located on secreted proteins and at the cell surface, and are therefore key determinants in molecular recognition events (Schjoldager et al., 2020).

Glycans exhibit tremendous structural diversity (Spiro, 2002). Unlike proteins, whose sequences can be predicted from gene sequences, the biosynthesis of glycans is not directly template‐driven; glycans are built, modified and trimmed by an extensive network of co‐expressed enzymes that are differentially expressed in cells and tissues, and can be affected by factors intrinsic and extrinsic to the cell. Glycan structures are therefore specific to various organisms, tissues, and cells, and the resulting structures can be highly heterogeneous, imparting additional complexity to the structural and functional properties of proteins (Schjoldager et al., 2020).

**FIGURE 1:**
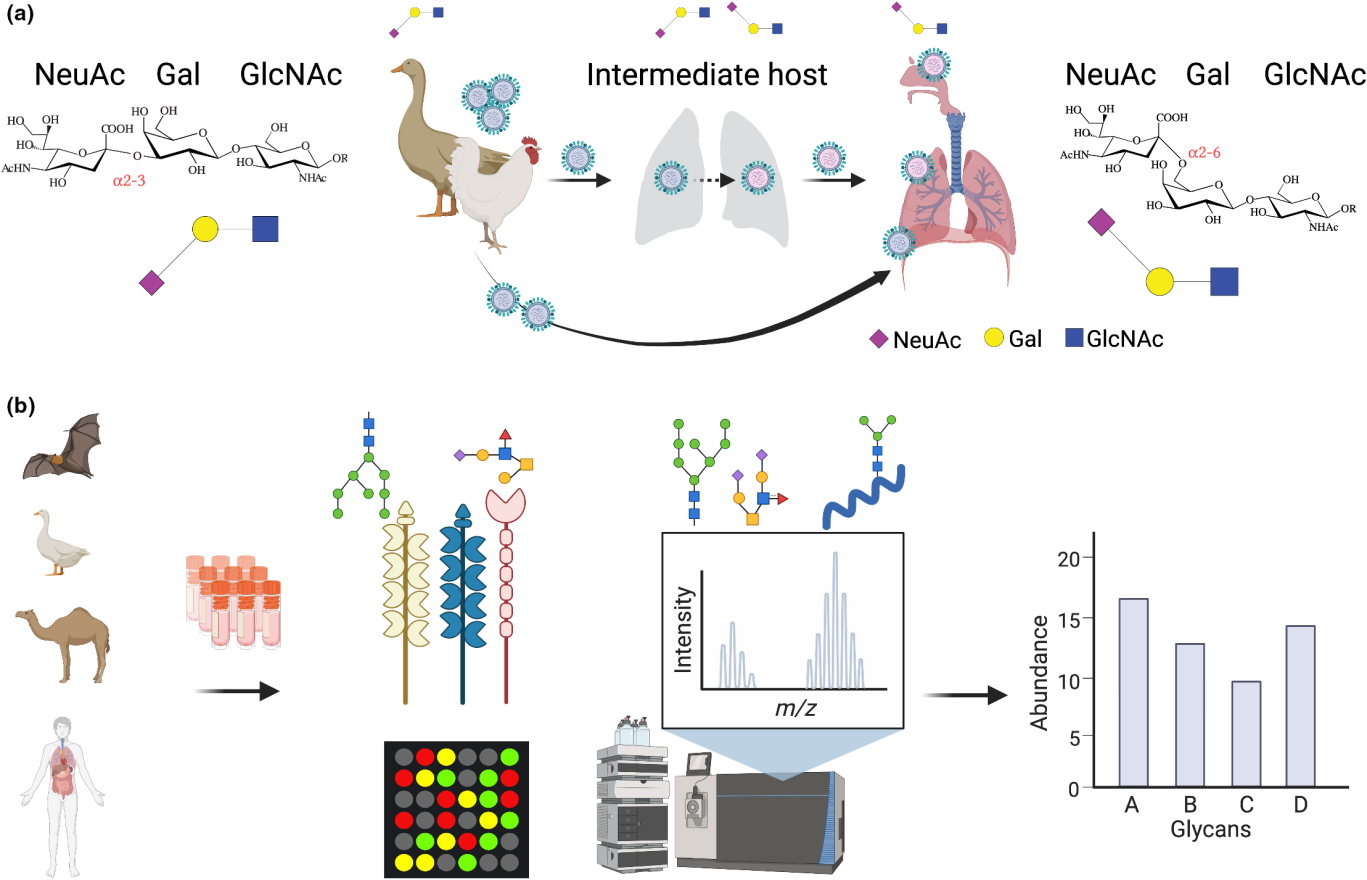
Glycosylation underlies the evolutionary shift towards zoonotic emergence of influenza. (a) When avian influenza makes the jump directly to humans, preferential binding to α2‐3‐linked sialic acid expressed in the lower lung leads to rarer infections that can be more severe but less transmissible. In intermediate hosts that express both α2‐3‐linked and α2‐6‐linked sialic acid, viruses can undergo an evolutionary shift that facilitates emergence in humans, who express α2‐6‐linked sialic acid in the upper respiratory tract. (b) An array of different tools can be used to study the glycosylation profiles of tissues, cells or proteins from a global level down to the glycan level. Figure created with BioRender (biorender.com)

Glycans act as receptors, coreceptors or attachment factors for numerous viruses (Thompson et al., 2019) including HIV, dengue (Raman et al., 2016), MERS‐CoV (Park et al., 2019), influenza (Shinya et al., 2006), and SARS‐CoV‐2 (Yang et al., 2020). For example, both avian and human‐adapted influenza viruses bind to glycans that terminate with sialic acid, but avian influenza preferentially binds to sialic acid with α2‐3‐linkages, while human‐adapted influenza prefers α2‐6‐linkages, which are expressed in the human upper respiratory tract (Kuchipudi et al., 2009; Shinya et al., 2006). When avian‐origin influenza lineages jump directly into humans, infections mostly become established in the lower lungs where cells express α2‐3‐linkages, leading to rare infections that are generally more severe but less transmissible (Stevens et al., 2006). Animal bridge hosts that can be infected by different influenza subtypes (including avian species) or express both types of sialic acid ‐ for example, swine ‐ provide an environment where viral lineages may directly undergo mutation to adapt to α2‐6‐binding (Thompson & Paulson, 2021), or where human and avian lineages cocirculate and undergo reassortment (Figure 1a). These hosts therefore provide an evolutionary stepping stone for avian lineages to switch to α2‐6‐binding, opening a transmission route for harmful zoonoses and producing more transmissible strains that pose epidemic or pandemic threats in humans (Chothe et al., 2017).

In cases like these, glycosylation is a key driver of host range and zoonotic risk, but one that is often neglected or folded into the “black box” of host‐virus interactions and evolutionary dynamics in systems that are less well‐characterized than influenza. However, the general importance of glycosylation in host‐pathogen interactions is well established (Suenaga & Arase, 2015; Watanabe et al., 2019). Given that potential differences in glycosylation presence and structure can have profound effects on molecular interactions, we suggest there is a clear need to measure the glycomes of potential hosts as part of broader efforts to describe viral ecology and emergence.

## 3 CHARACTERIZING GLYCANS AT DIFFERENT SCALES

The inherent structural complexity and heterogeneity of glycans across species, individuals, organ systems, tissues, and even time and space make them an analytically challenging subject. Additionally, and in contrast to other biomolecules, they can have extraordinarily high structural complexity due the variety of monosaccharide building blocks and the multiple ways they can attach to each other, both in bond configuration (α and β) and in the positions of the intersaccharide linkages within the molecules (Cummings, 2009). Nevertheless, a number of techniques can be used individually or in combination to characterize protein glycosylation, with mass spectrometry being a powerful and widely used tool that can be incorporated at various levels (Figure 1b). Generally, the overarching aim of glycosylation analyses is to deduce one or more of the following: the monosaccharide composition of the glycans, the order and branching of monosaccharides in a glycan, the types of glycosidic linkages and monosaccharide anomericity, or the location of the glycosylation sites on a protein. The functional roles of glycans can also be assessed by measuring noncovalent interactions between specific proteins and glycans. A global view can be obtained from antibody or lectin binding to selectively identify glycan epitopes or motifs in tissues, cells and proteins. Lectins can be used in array‐based platforms (Gao et al., 2019) enabling high‐throughput analyses with the caveat that they do not provide comprehensive structural information. The most effective way to obtain structural details of glycans is to use a glycomic workflow whereby the glycans are chemically or enzymatically released from glycoproteins. The released glycans are typically chemically labelled with fluorescent tags and analysed by liquid chromatography or capillary electrophoresis with a mass spectrometer used for enhanced detection (Everest‐Dass et al., 2018). These methods for studying the glycome can provide monosaccharide composition and sequence information, and at times, linkage position. Coupling these analytical techniques with enzymes that cleave specific monosaccharide linkages provides additional precise structural information.

A limitation of glycomic approaches is that protein‐ and site‐specificity is lost with glycan release. Nevertheless, these techniques are the most powerful for providing the basic information about glycan structure that will probably form the basis of future predictive models (see below). This is because the same glycan structures or epitopes can often be found on many different sites and proteins from the same cell, because they share the same glycan biosynthetic pathways. In addition, a key benefit of glycomics, compared to proteomics or glycoproteomics (the study of proteins and glycosylated proteins, respectively), is that analysis of released glycans does not typically require prior knowledge of the genome. Although glycan biosynthesis is nontemplate driven and millions of possible glycan structures can be predicted (Cummings, 2009), there are comparatively few glycan structures actually observed on glycoproteins (Werz et al., 2007), and these can be predicted or de novo structurally determined without knowledge of the genome (Kellman & Lewis, 2021). If the annotated genome is available, genomic and phylogenetic profiling of glycan metabolism enzymes can greatly assist or validate glycomic approaches (McVeigh et al., 2018), especially where the glycan profiles from species contain structures that have not yet been defined (West et al., 2021). Furthermore, mass spectrometry glycoproteomics can also be used to identify and measure peptides with attached glycans. In this case, fine structural detail of the glycan structure is lost but the site of attachment and the level of site occupancy is retained.

## 4 BUILDING GLYCOSYLATION INTO PREDICTIVE SCIENCE

**FIGURE 2:**
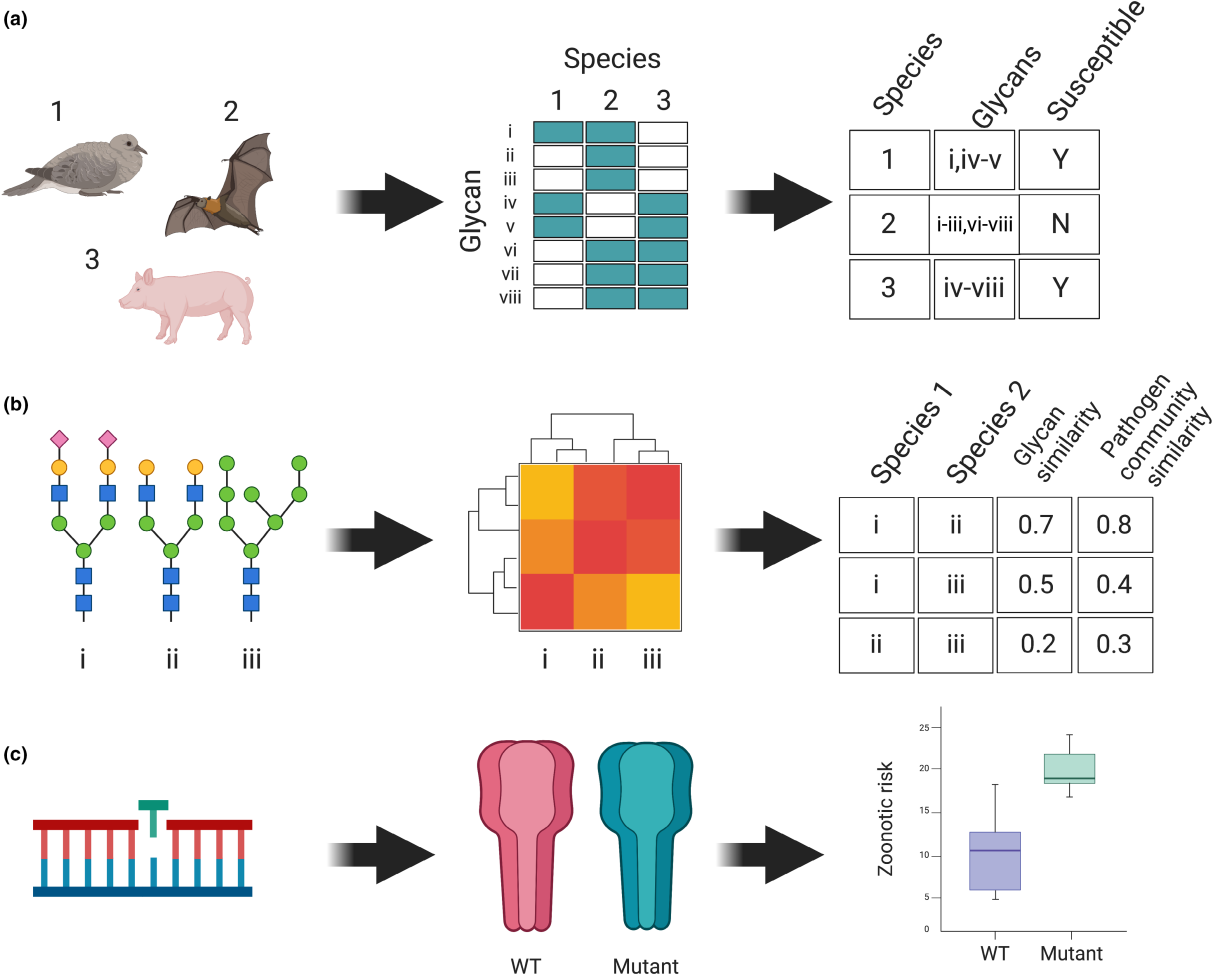
Adding glycosylation to microbiology‐smart modelling. (a) Different animals have different combinations of glycans, which may help unpack how specific glycans contribute to susceptibility to a given pathogen. In this example, species 2's lack of glycans iv and v could explain its lack of susceptibility to the pathogen. (b) When the mechanism is understood in better detail, the glycosylation of a single structure (e.g., the ACE2 receptor) might help predict cross‐species transmission potential for a specific virus (e.g., SARS‐CoV‐2) ‐ if the structural similarity of a given glycan can be converted into machine‐readable features. (c) Understanding mechanisms in greater detail may improve other kinds of predictions about cross‐species transmission: for example, mutations in the haemagglutinin structure of influenza viruses limit their binding efficiency to the glycosylation of human sialic acid receptors, allowing prediction of the zoonotic potential of specific influenza A strains based on a few point mutations. Figure created with BioRender (biorender.com)

Understanding the landscape of host glycosylation might help scientists build better predictive tools to understand the broader rules of viral cross‐species transmission or even the special case of zoonotic risk. This could be accomplished in a number of ways, most of which are untested. Glycosylation could be represented as data in several ways, ranging from simple (e.g., the presence or absence of a specific set of glycan structures) to complex (generating quantitative features using graph representations of the glycan structure [Alonso et al., 2018]). These data can then be used several ways. For example, recent attention on SARS‐CoV‐2's use of the ACE2 receptor has sparked the development of models that predict host susceptibility based on receptor sequences (Fischhoff et al., 2021), but glycosylation is a missing element; incorporating glycan structures as receptor or coreceptor “metadata” might help researchers better understand viral attachment (Figure 2a). For instance, sialic acid and heparan sulphate are key cell surface glycans that are coreceptors for SARS‐CoV‐2 (Clausen et al., 2020; Nguyen et al., 2021). Conversely, from the host perspective, specific glycans may promote viral entry as is the case for SARS‐CoV‐2, where site‐specific glycans of ACE2 have been implicated in receptor‐viral binding (Zhao et al., 2020). Thus similar glycosylation might help explain pathogen sharing between different animals (Figure 2b), and (according to some preliminary evidence [Burkholz et al., 2021]) might even help unpack some of the microbiology inside the black box “phylogenetic distance effect” that broadly structures the viral sharing network (Albery et al., 2020). If viral glycosylation helps evade host immune system detection, these dimensions of similarity may help explain how particular bridge host “stepping stones” are possible, including in cases of zoonotic emergence (during early characterization, glycan motifs or structures can even be proactively searched to identify homology in human and animal hosts, and linked to viruses where glycan‐binding preferences have been established). Similarly, understanding the role of viral mutations that alter the viral surface glycoproteins (Figure 2c) may lead to more targeted insights about how glycosylation relates to zoonotic risk (as in the example of influenza and sialic acid; Figure 1a).

Most of these examples are still hypothetical, but in limited cases, these types of model‐based exploration have shown tremendous promise. For example, a recent study used graph representations of glycans and multiple kinds of advanced machine learning (graph convolutional neural networks and natural language models) to predict host identity and glycan immunogenicity, and was able to predict influenza and rotavirus binding affinity for host receptors from different species (Burkholz et al., 2021). Studies like these are exciting proofs‐of‐concept and point to the idea that feature representations of glycans may eventually be useful as part of a broader palette of cell‐ and virus‐level trait predictors used to make even more advanced (and crucially, microbiology‐driven) machine learning or network models.

In order to power these kinds of approaches, more data is needed about the “global glycome.” While the human glycome is well studied (Jia et al., 2020), the glycomes of animal reservoirs are severely understudied ‐ a major problem when, for example, host range is extensive (e.g., influenza A virus can infect captive and wild animals including birds, dogs, cats, pigs, horses, bats, seals and even some reptiles [Short et al., 2015]). Where existing glycomic data sets are unavailable, transcriptomics coupled with experimentally‐defined and predicted glycan biosynthetic pathways could help fill the gaps (Dworkin et al., 2022; Kellman & Lewis, 2021) while existing glycomic data sets can be used to train models. When expanding existing data sets, wild reservoir populations should be a priority for experimental glycosylation analyses, particularly those that are endemically infected, at the human‐animal interface and those likely to act as an intermediate “mixing vessel” for cross‐species transmission. Tissues, cells and fluids that are the routes of entry for infectious agents or are predicted to be involved in tropism and systemic spread should be a focal point of analysis. These may include the mucosal epithelial tissue of the gastrointestinal, urogenital and respiratory tracts and cells of the skin, lymphatic system and blood vessels. The types of data to be incorporated from these sample types could range from global glycome analyses to precise glycan structure and site occupancy information. Ultimately, the types of experiments conducted will be determined by the capabilities of the laboratory. In depth protocols are available for global structural (Jensen et al., 2012; Li et al., 2020) and protein‐ and site‐specific (Hart & Wells, 2021; Kolarich et al., 2012; Oliveira et al., 2021; Remoroza et al., 2021; Riley et al., 2020) characterization of glycans. Given the versatility of liquid chromatography mass spectrometry to study glycosylation, this approach seems most accessible with costs predicted to be in the low to medium range depending on the level of structural characterization achieved. With unlimited sample availability and technical resources, multiple tissues from multiple species could be characterized and this data used for modelling. Tissue types may be limited due to the requirements for lethal or nonlethal sampling, the ability to dissect out specific tissues, or even the capacity to sample all species, though many field researchers have suitable samples already collected and in storage. As with most fieldwork, sample availability will be dictated by each situation, but a balance should be struck between accessible biofluids (such as blood) and relevant tissues (such as lung tissue for respiratory viruses). On the other hand, given the ever‐growing frequency of glycomic and glycoproteomic techniques (lectin‐arrays or mass spectrometry‐based), it is possible that the analytical work could be accomplished collaboratively at low to medium cost as part of broader comparative zoology and viral ecology. Critically, depositing the data sets generated by this work into existing public repositories like GlycoPOST and GlyTouCan, which is becoming standard practice in the glycobiology field (Rojas‐Macias et al., 2019), will allow their value to grow exponentially for comparative and predictive research.

Recent viral epidemics and pandemics have highlighted a need for increased surveillance at the animal‐human interface and forward planning of biochemical countermeasures (Rabozzi, 2020; Lurie et al., 2020; Munir et al., 2020). Characterizing the global glycome will help microbiologists and ecologists understand the broader dynamics of viral ecology, and these data could also easily be applied to understanding other pathogens. Moreover, as work during the COVID‐19 pandemic has highlighted, understanding glycosylation as a viral phenotype is a key part of understanding pathogenesis and developing effective countermeasures (Shiliaev et al., 2021; Uraki & Kawaoka, 2021), and we suggest that building more glycomics into viral surveillance is a feasible, cost‐effective, and impactful way to expand the body of basic science that forms the basis for epidemic preparedness.

## AUTHOR CONTRIBUTIONS

Colin J. Carlson and Benjamin A. Neely conceptualized the perspective. All authors contributed to the writing and review of the present study.

## CONFLICT OF INTEREST

The authors declare no competing interests.

## Data Availability

Data sharing not applicable to this article as no data sets were generated or analysed during the current study.
